# 

**Published:** 1883-09

**Authors:** 


### CONTENTS.

### COMMUNICATIONS.

Address of Dr. J. G. Fredericks ........ 409
A Few Practical Observations on the Treatment of the Pulp ........ 412
American Dental Association ........ 416

### PROCEEDINGS.

Transactions of the Association of State Boards of Dental Examiners. 419
Questions Prepared and Adopted by the State Boards of Dental Examiners........ 428
A Synopsis of the Proceedings of the American Dental Association 433

### SELECTIONS.

Medicine as Practiced by Animals........ 444
Academy of Medicine in Ireland........ 448
Something Practical about Micrococci........ 450
Lister and the Spray........ 451
A Substitute for Celluloid ........ 451
Billroth's Clinic in 1882........ 452
Selections from a Clinical Lecture........ 453
Severe Hæmorrhage after Tooth-Extraction, Treated by Transfusion 454
Ether Spray in Facial Neuralgia........ 455
Danger of Spreading Disease by Books ........ 455
The Bi-Chromate Battery ........ 456
Trichlorophenate of Lime ........ 456
Another Use for Carbolic Acid........ 457
Remarkable Arrest of Development........ 457
Sounds from the Consulting Room ........ 458
To Remove Fish-Bones from the Throat ........ 458

### EDITORIAL.

Danger—Carelessness ........ 459

